# Vitamin D Receptor Regulates Liver Regeneration After Partial Hepatectomy in Male Mice

**DOI:** 10.1210/endocr/bqae077

**Published:** 2024-07-04

**Authors:** Harendran Elangovan, Rebecca A Stokes, Jeremy Keane, Sarinder Chahal, Caroline Samer, Miguel Agoncillo, Josephine Yu, Jennifer Chen, Michael Downes, Ronald M Evans, Christopher Liddle, Jenny E Gunton

**Affiliations:** The Centre for Diabetes, Obesity and Endocrinology Research (CDOER), The Westmead Institute for Medical Research, The University of Sydney, Sydney, NSW 2145, Australia; The Centre for Diabetes, Obesity and Endocrinology Research (CDOER), The Westmead Institute for Medical Research, The University of Sydney, Sydney, NSW 2145, Australia; The Centre for Diabetes, Obesity and Endocrinology Research (CDOER), The Westmead Institute for Medical Research, The University of Sydney, Sydney, NSW 2145, Australia; The Centre for Diabetes, Obesity and Endocrinology Research (CDOER), The Westmead Institute for Medical Research, The University of Sydney, Sydney, NSW 2145, Australia; Pharmacogenomics and Personalized Therapy Unit, Geneva University Hospitals, Geneva 1205, Switzerland; The Centre for Diabetes, Obesity and Endocrinology Research (CDOER), The Westmead Institute for Medical Research, The University of Sydney, Sydney, NSW 2145, Australia; The Centre for Diabetes, Obesity and Endocrinology Research (CDOER), The Westmead Institute for Medical Research, The University of Sydney, Sydney, NSW 2145, Australia; The Centre for Diabetes, Obesity and Endocrinology Research (CDOER), The Westmead Institute for Medical Research, The University of Sydney, Sydney, NSW 2145, Australia; Gene Expression Laboratory, Salk Institute for Biological Studies, La Jolla, CA 92037-1002, USA; Gene Expression Laboratory, Salk Institute for Biological Studies, La Jolla, CA 92037-1002, USA; Storr Liver Centre, The Westmead Institute for Medical Research, The University of Sydney, Sydney, NSW 2145, Australia; Faculty of Medicine and Health, The University of Sydney, Sydney, NSW 2145, Australia; The Centre for Diabetes, Obesity and Endocrinology Research (CDOER), The Westmead Institute for Medical Research, The University of Sydney, Sydney, NSW 2145, Australia; Faculty of Medicine and Health, The University of Sydney, Sydney, NSW 2145, Australia; Department of Diabetes and Endocrinology, Westmead Hospital, Sydney, NSW 2145, Australia

**Keywords:** vitamin d receptor, hepatocyte, liver regeneration, cell cycle, bile acids

## Abstract

Vitamin D signals through the vitamin D receptor (VDR) to induce its end-organ effects. Hepatic stellate cells control development of liver fibrosis in response to stressors and vitamin D signaling decreases fibrogenesis. VDR expression in hepatocytes is low in healthy liver, and the role of VDR in hepatocyte proliferation is unclear. Hepatocyte-VDR null mice (hVDR) were used to assess the role of VDR and vitamin D signaling in hepatic regeneration.

hVDR mice have impaired liver regeneration and impaired hepatocyte proliferation associated with significant differential changes in bile salts. Notably, mice lacking hepatocyte VDR had significant increases in expression of conjugated bile acids after partial hepatectomy, consistent with failure to normalize hepatic function by the 14-day time point tested. Real-time PCR of hVDR and control livers showed significant changes in expression of cell-cycle genes including cyclins D1 and E1 and cyclin-dependent kinase 2. Gene expression profiling of hepatocytes treated with vitamin D or control showed regulation of groups of genes involved in liver proliferation, hepatitis, liver hyperplasia/hyperproliferation, and liver necrosis/cell death. Together, these studies demonstrate an important functional role for VDR in hepatocytes during liver regeneration. Combined with the known profibrotic effects of impaired VDR signaling in stellate cells, the studies provide a mechanism whereby vitamin D deficiency would both reduce hepatocyte proliferation and permit fibrosis, leading to significant liver compromise.

Vitamin D receptor (VDR) mediates the direct actions of vitamin D in cells and tissues. There is mounting evidence to suggest a role for the VDR-endocrine axis in the modulation of hepatic pathophysiology (1-3). Vitamin D deficiency associates with poorer outcomes in a range of liver diseases (1, 4), including responses to hepatitis B and hepatitis C viral infection, and metabolic associated fatty liver disease.

Impaired vitamin D action is associated with increased fibrosis in many tissues (1, 3, 5-7). We have previously reported that lack of vitamin D signaling resulting from genetic inactivation of VDR leads to liver fibrosis and spontaneous cirrhosis in some mice from age of 6 months onwards (3). VDR in hepatic stellate cells controls the TGF-β “switch,” whereby TGF-β signaling without VDR signaling promotes fibrosis. TGF-β signaling with activation of VDR by vitamin D promotes liver recovery (3).

In contrast to stellate cells, normal hepatocytes express very low levels of VDR (8). Thus, the prevailing dogma is that VDR regulates hepatocyte function and liver repair through effects on nonparenchymal liver cells, such as the hepatic stellate cell (8). However, hepatocytes do express low levels of VDR. In other tissues, VDR expression usually increases with injury, suggesting the possibility of a role in regeneration or recovery.

Whether VDR in hepatocytes plays a role in recovery following liver injury and subsequent hepatic regeneration is unclear. The roles of vitamin D in metabolism in other tissues are complex (9), including potentially confounding effects on calcium and phosphate handling. To examine this question, without the complexity of other tissues, or effects on other liver cell types, hepatocyte-specific VDR null mice (hVDR) were bred, and liver regeneration was studied using a 2/3 partial hepatectomy model (10). The hypothesis was that hVDR mice would show an impaired regenerative response after partial hepatectomy.

## Materials and Methods

### Animals and Ethics Approval

**Figure 1.**
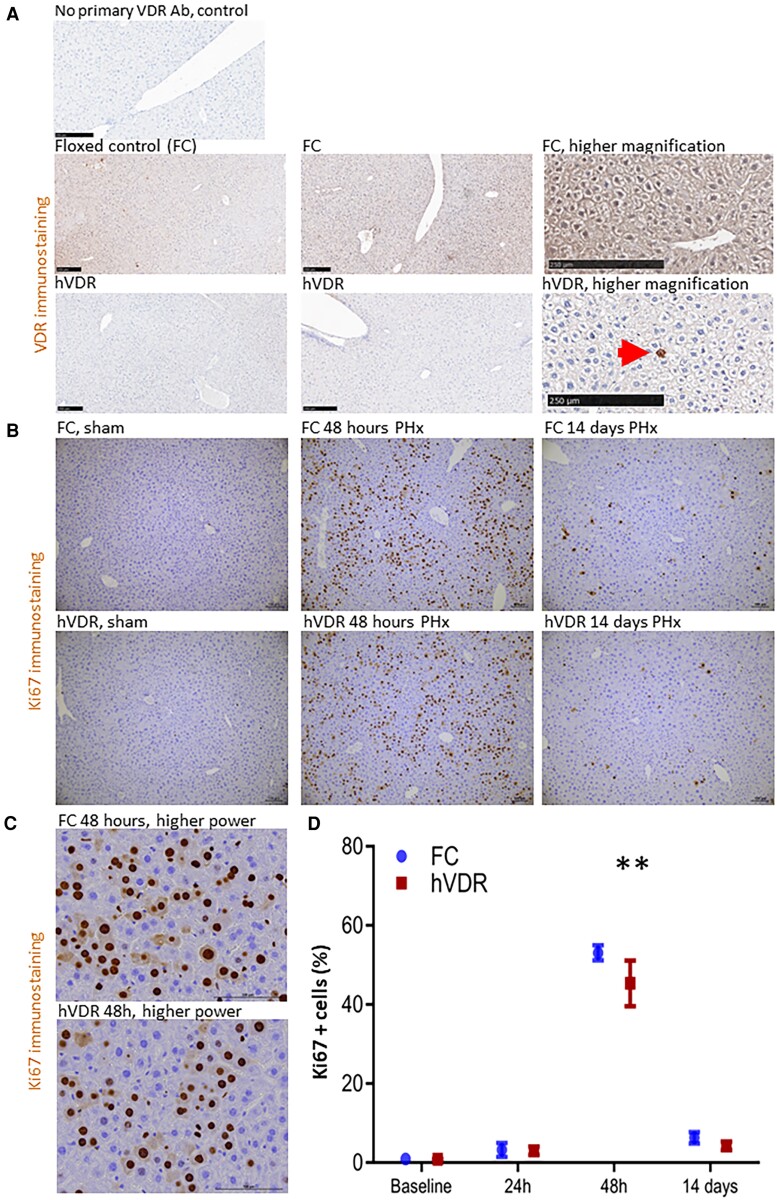
Liver histology in floxed control (FC) and hVDR mice. (A) VDR immunostaining and (B) Ki67 immunostaining. (C) Higher magnification of Ki67 immunostaining and (D) quantification by ImageJ. **P < .01. *P* = .057 at 14 days.

hVDR mice and their floxed control (FC) littermates were bred using the Cre-LoxP system. Mice with albumin promoter-driven expression of Cre-LoxP were bred with floxed VDR mice, generating mice with VDR deletion in albumin-expressing cells. For experimental mice, the male parent was VDR fl/fl, Cre-positive, and the female parent was VDR fl/fl, Cre negative. Floxed VDR mice were developed as previously described (11) by Geert Carmeliet. Figure 1A shows histology from FC and hVDR livers. All animals were bred on a C57Bl/6 background and obtained from the Animal Bio-resources Facility (Moss Vale, NSW, Australia). Studies were approved by Western Sydney Local Health District AEC (approval 4264). Animals were housed in a specific pathogen-free facility with 12-hour light and dark cycles and provided ad libitum access to standard rodent chow and water.

Partial hepatectomy to remove 2/3 of the liver was performed as described by Mitchell and Willenbring (10). It was performed on male mice during the morning hours of 7:30 to 11:30 Am. In brief, mice were anesthetized with isoflurane (3%-5%) with supplemental oxygen (1 L/min), mice were shaved, the skin was sterilized, and the peritoneum was accessed via a midline laparotomy. The left lateral and median lobes (including the gallbladder), which altogether account for approximately 70% of murine liver mass, were excised in 2 separate ligations (10). The resected lobes were blotted gently on sterile gauze, then weighed and divided baseline assessments including formalin fixation for histology and snap freezing. The abdomen was closed in layers using running 4-0 sutures. Mice were provided a single dose (0.08 mL/100 g) of buprenorphine IP at the end of the surgery as postoperative analgesia. Mice were place under a heating lamp until they were self-righting and then single-housed for the next 2 to 3 days. Buprenorphine was readministered as needed in the following 3 days if any mice showed signs of discomfort. Sham-operated mice were anesthetized, shaved, and had their abdominal skin sterilized. Further sham procedures were not permitted by the Animal Ethics Committee.

At the designated study endpoint (1, 2, or 14 days), mice were euthanized by carbon dioxide and blood was collected by cardiac puncture with 5 µL of EDTA. The right and caudate lobes were freed from any attachments, rinsed in saline, gently dried on sterile gauze, and then weighed. The regeneration samples were also apportioned for snap-freezing in liquid nitrogen or fixed in formalin for histological study. Collected blood was centrifuged at 16 000*g* and the plasma was stored at −20 °C for subsequent analyses. Day 14 was chosen to assess near-complete regeneration and then days 1 and 2 were carried out in new mice to examine potential mechanisms for the effects observed at 14 days.

For the 1 and 2 day times, mice were 8 to 9 weeks at study commencement (N = 11-12 per group for 1 day and n = 7-8 per group at 2 days). For the 14-day endpoint, mice were studied at 18 to 20 weeks of age at the start of the study (N = 11 per group). Mice that did not survive surgery are excluded from those counts and data were not included in analysis (n = 8 across all groups and all times).

### Gene Expression

For real-time PCR, RNA was extracted from liver sections using the RNeasy Kit (QIAGEN) according to the manufacturer's instructions. Equal amounts of RNA were then reverse transcribed using the Maxima First Strand c-DNA synthesis kit (Thermo Scientific) and analyzed by quantitative PCR using the SYBR Green PCR master mix preparation (Applied Biosystems, Warrington, UK). Reactions were amplified and quantified using an ABI 7900 light cycler (Applied Biosystems) and normalized to the expression of Cyclophilin, which did not differ between groups and thus was employed as a house-keeping gene. Primers were designed using the Primer BLAST software (National Library of Medicine, USA) and obtained from Invitrogen. Primer sequences used in this study are listed in Table 1. Data are shown as fold-change vs day 0 floxed controls.

**Table 1.** Primer sequences

Gene	Forward primer	Reverse primer
B-Cell Lymphoma 2 (*Bcl2*)	5′-tctgaaggattgatggcaga-3′	5′-catcagccacgcctaaaagt-3′
BCL-2-associated X protein (*Bax*)	5′-tgcagaggatgattgctgac-3′	5′-gatcagctcgggcactttag-3′
Cyclophilin	5′-tggaccaaacacaaacggttcc-3′	5′-acattgcgagatggggtag-3′
Cyclin-dependant kinase 2 (*Cdk2)*	5′-acaggactccaaaggctct-3′	5′-aaattcatggatgcctctgc-3′
Cyclin-dependant Kinase 4 (*Cdk4*)	5′-actctgaagccgaccagttg-3′	5′-ccagactcctccatctctgg-3′
Cyclin D1 (*Ccnd1)*	5′-cacaacttctcggcagtcaa-3′	5′-agtgcgtgcagaaggagatt-3′
Cyclin E1 (*Ccne1*)	5′-gagcaagcgccatctgtaac-3′	5′-tgtgcgaagtctataagctcca-3′
Cdk inhibitor 1 (*p21*)	5′aggcggctaccgtcctacta-3′	5′-gccttagccctcactgtggtg-3′
Cdk inhibitor 1B (*p27*)	5′-tctgacgagtcaggcatttg-3′	5′-cagaatcataagcccctgga-3′

### Measurement of Bile Acids

Bile acids were measured as previously described in detail in Fu et al (12). Briefly, 20 µL of mouse plasma was protein precipitated with 80 µL of ice-cold acetonitrile containing 3.28 ng of deuterated cholic acid (2, 2, 4, 4-d4 cholic acid) as an internal standard. The samples were vortexed for 1 minute and centrifuged at 10 000 rpm for 10 minutes at 4 °C. Supernatants were evaporated under vacuum at room temperature and reconstituted in assay mobile phase and transferred to a 96-well plate for analysis. A Nextera ultra-performance liquid chromatography system (Shimadzu, Kyoto, Japan) was used in combination with a Q-TRAP 5500 Mass Spectrometer (AB Sciex, Toronto, Canada) with Analyst Software 1.6.2 (Kakiyama et al, 2014). Chromatographic separations were performed with an Acquity (Water, Milford, MA) ultra-performance liquid chromatography BEH C18 column (1.7 µm, 2.1 × 100 mm). The temperatures of the column and auto sampler were 65° and 12°, respectively. Sample injection was 1 mL. The mobile phase consisted of 10% acetonitrile and 10% methanol in water containing 0.1% formic acid (mobile phase A) and 10% methanol in acetonitrile 0.1% formic acid (mobile phase B) delivered as a gradient: 0 to 5 minutes mobile phase B held at 22%; 5 to 12 minutes mobile phase B increased linearly to 60%, 12 to 15 minutes mobile phase B increased linearly to 80% and 15 to 19 minutes mobile phase B constant at 80% at a flow rate of 0.5 mL/min. The mass spectrometer was operated in negative electro-spray mode working in the multiple reaction mode. Operating parameters were Curtain gas 30 psi; Ion spray voltage 4500 V; Temperature 550 °C; Ion Source Gas 1 60 psi; Ion Source Gas 2 65 psi. Transition multiple reaction modes, declustering potential entrance potentials, and collision cell exit potentials were optimized using the Analyst software. Dwell times were 25 ms.

Plasma levels of the liver enzymes alanine aminotransferase (ALT) and aspartate aminotransferase (AST) were assessed using a Cholestech LDX analyser (Cholestech, CA). Because of the limited availability of cartridges, measurement of liver enzymes was restricted to blood collected from mice sacrificed at day 14.

### Histology

Liver specimens were fixed in 10% neutral buffered formalin and subsequently paraffin embedded. Sections were cut using a microtome (Leica) at 5-μm thickness and stained with hematoxylin and eosin, or Sirius red according to standard protocols (3, 13, 14). Sections were evaluated by at least 2 observers blinded to the genotype of the animals.

For studies of VDR, vitamin D receptor protein was immune-stained used the D6 antibody RRID:AB_628040 and the goat anti-mouse peroxidase antibody RRID:AB_2617137. For cellular proliferation, liver sections were immunostained for Ki-67, a nuclear protein highly specific for cells that have entered the cell cycle. In brief, sections were subjected to a standard dewaxing and progressive ethanol rehydration protocol. Deparaffinized sections then underwent heat-induced epitope retrieval in a pressure cooker using a pH 9.0 tris/EDTA target retrieval solution (S2367) (Dako Corp, Carpentaria, USA). Following peroxidase and protein blocking (Dako) for 10 and 30 minutes, respectively, sections were incubated at room temperature with a primary rabbit monoclonal antibody against Ki-67 (SP6 clone, Thermo Scientific, Cat: MA5-14520, RRID:AB_10979488) (1:200 dilution using Dako antibody diluent) for 60 minutes. This was accompanied by treatment with a secondary anti-rabbit horseradish peroxidase-conjugated antibody (Envision Rabbit, RRID:AB_2630375) for 30 minutes and signal detection using a 3, 3-diaminobenzidine chromogenic substrate solution for 10 minutes. Optimal antigen retrieval protocols, blocking agents/steps, antibody choice, dilutions, and incubation times were determined on the basis of pilot studies conducted during the course of this project. Finally, sections were counterstained using a hematoxylin and ethanol dehydration process and subsequently cover-slipped with aqueous mounting fluid (Dako). Immunostained slides were scanned by Nanozoomer. Positively labelled cells were counted in entire sections using ImageJ.

For assessment of cell size, images of at least 8 different slides from at least 6 mice per group were assessed. A square of 250 µm × 250 µm was assessed for each slide and the number of cells counted to give average cell cross-sectional area.

Rat hepatocytes were isolated from Sprague Dawley rats by collagenase perfusion in situ. Hepatocytes were plated on collagen and cultured in Williams W Medium. After 48 hours, cells were treated for 24 hours with 1,25(OH)2-vitamin D3 at 10 nmol/L. RNA was extracted and prepared for hybridization to Illumina Rat-Ref12 Microarrays. Arrays were analyzed and differential gene expression was assessed with Illumina GenomeStudio software.

A publicly available dataset (GSE138376) that examined HepG2 cells transfected with VDR and treated with control or vitamin D (15) was obtained and analyzed.

### Statistics

**Figure 2.**
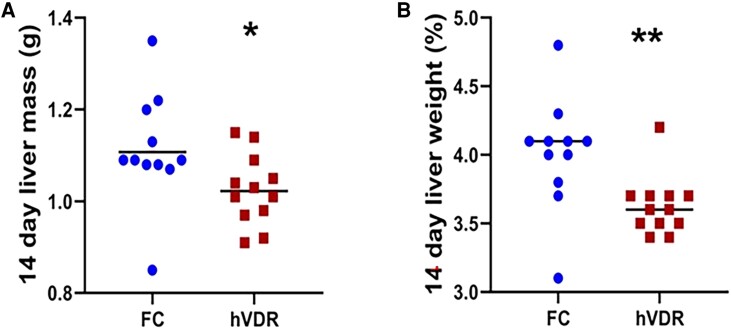
Liver mass 14 days after partial hepatectomy. (A) Absolute liver weights in FC and hVDR mice. (B) Liver weight as a proportion of body mass. Data show individual values for mice; the line indicates mean. *P < .05, **P < .01. Unpaired *t*-tests with Welch's correction (Prism).

**Figure 3.**
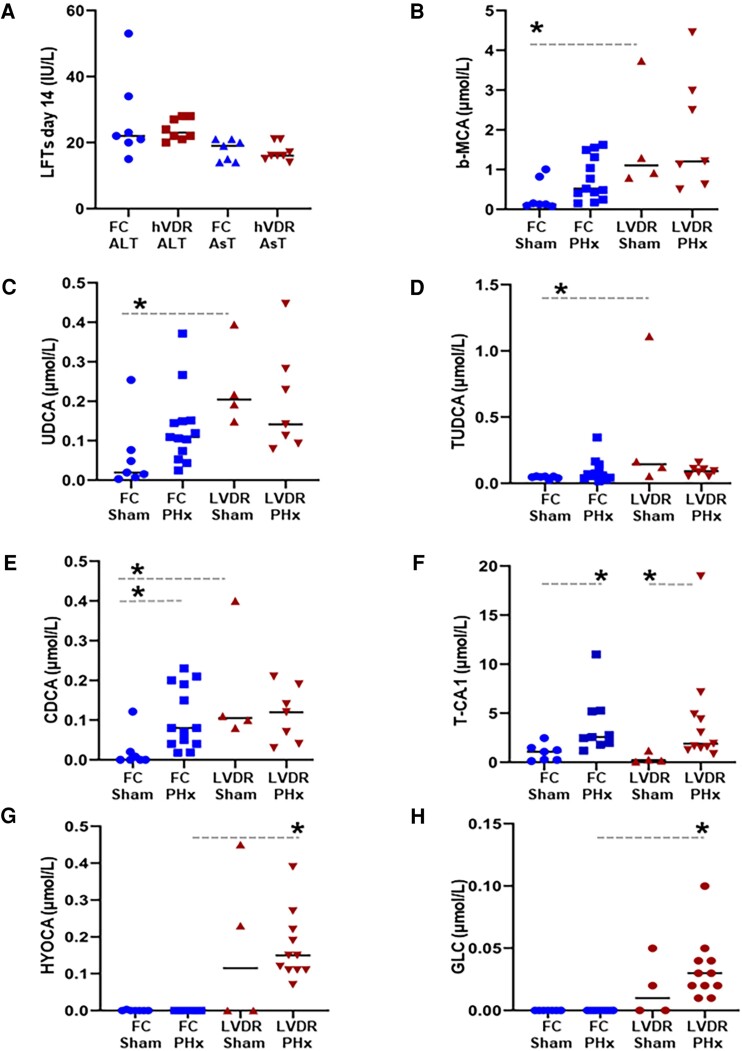
Liver function in floxed control (FC) and hVDR mice at day 14. (A) Liver function tests (B-H) Serum bile acids in FC and hVDR mice. bMCA, tauro-β-muricholic acid; CDCA, chenodeoxycholate; GLC, glycolithocholate; HYOCA, hyocholic acid; T-CA1, taurocholic acid; TUDCA, tauroursodeoxycholate, UDCA, ursodeoxycholate. *P < .05 for indicated comparison (ANOVA and post hoc testing with correction for multiple comparisons).

Statistical significance was assessed using Excel for Student *t*-tests, or GraphPad Prism 8, or SPSS version 25. Bile acids were not normally distributed and were assessed by Kruskal-Wallis testing with Dunn correction for multiple comparisons. A *P* value of <.05 after correction for multiple comparisons was considered significant. Figures show mean ± SEM unless otherwise indicated (for example Figs. 2 and 3 show all values).

## Results

hVDR mice were bred using the Cre-Lox system, and breeding floxed VDR mice, and albumin-Cre transgenic mice. The hVDR mice had slightly lighter bodyweights than their littermate floxed controls in the 8- to 9-week age groups (23.2 ± 0.5 g vs 24.7 ± 0.6 g in FC, *P* = .043). This difference was no longer apparent at 18 weeks of age (30.1 ± 0.5 g in hVDR vs 29.9 ± 0.5 g). Mice otherwise did not appear different before surgery at either age.

Resected liver weight at partial hepatectomy was identical in the young group (0.7 ± 0.02 g vs 0.7 ± 0.02 g) but, with the lower body weight, was greater when expressed as a proportion of body weight (3.03 ± 0.07% of body weight in hVDR vs controls at 2.83 ± 0.05%, *P* = .02). This was not significant in the older mice (2.73 ± 0.03% in hVDR vs 2.69 ± 0.05%, *P* > .6). There were no differences in liver histology in hVDR mice in the baseline resected samples.

One day (24 hours) after resection, there were no significant differences in residual liver weight (0.46 ± 0.02 g vs 0.43 ± 0.04 g). At 48 hours after partial hepatectomy (PHx), there was also no significant difference in residual liver weight (0.51 vs 0.51 g) between genotypes, although the liver mass was significantly larger in both genotypes compared to 24 hours (2-way ANOVA *P* = .006 for day, *P* = .455 for genotype).

VDR histology in controls and hVDR mice is show in Fig. 1A. Histological assessment of Ki67 positivity showed the expected very low hepatocyte proliferation in sham-control mice that had not undergone surgery both for FC and hVDR at 48 hours (Fig. 1B). In contrast, proliferation was substantially increased in FC PHx mice at 48 hours to 57.1 ± 3.2% of all cells (Fig. 1B; higher magnification images in Fig. 1C) but this increase was significantly blunted in hVDR PHx mice (40.0 ± 6.5%, *P* = .002 vs controls, Fig. 1D). There was a trend to decreased proliferation in hVDR mice at 14 days (Fig. 1D, 3.6 ± 1.3 vs 2.5 ± 0.9% in hVDR, *P* = .057) but this did not achieve statistical significance.

Consistent with the decreased proliferation seen at 48 hours, the absolute weight of the regenerated liver was reduced in hVDR mice 14 days postsurgery (1.025 ± 0.023 g vs 1.11 ± 0.039 g in FC, *P* = .048, Fig. 2A). Expressed as percentage of body weight, the regenerated liver was also significantly lighter in hVDR mice (3.6 ± 0.2% vs 4.0 ± 0.4% of body weight, *P* = .0048, Fig. 2B).

To assess markers of liver injury after partial hepatectomy, ALT and AST and plasma bile acids were measured at 14 days. There were no significant differences in ALT or AST (Fig. 3A). Bile acids are synthesized by the liver, enterohepatically recirculated, and increased in response to liver injury (16). They were measured by mass spectrometry to assess hepatic metabolism. In sham-operated mice, hVDRs have higher tauro-β-muricholic acid (*P* = .048), ursodeoxycholate (*P* = .014), and tauroursodeoxycholate (*P* = .012) (Fig. 3B-3D) compared to floxed controls. These bile acids were not significantly different between genotypes after partial hepatectomy (Fig. 3B-3D).

Chenodeoxycholate (CDCA), which is a ligand for the farnesoid X receptor (FXR), was higher in hVDR than FC in sham mice, and FC showed significant increases in CDCA after partial hepatectomy (Fig. 3E). There was no increase in CDCA in hVDR mice (Fig. 3E).

The surgery of partial hepatectomy resulted in increased taurocholic acid in both FC and hVDR mice (Fig. 3F, *P* = .024 for both compared to shams) without any differential effect of genotype.

Partial hepatectomy caused significant increases in hyocholic acid and glycolithocholate only in hVDR mice (Fig. 3G and 3H, both *P* < .05).

**Figure 4.**
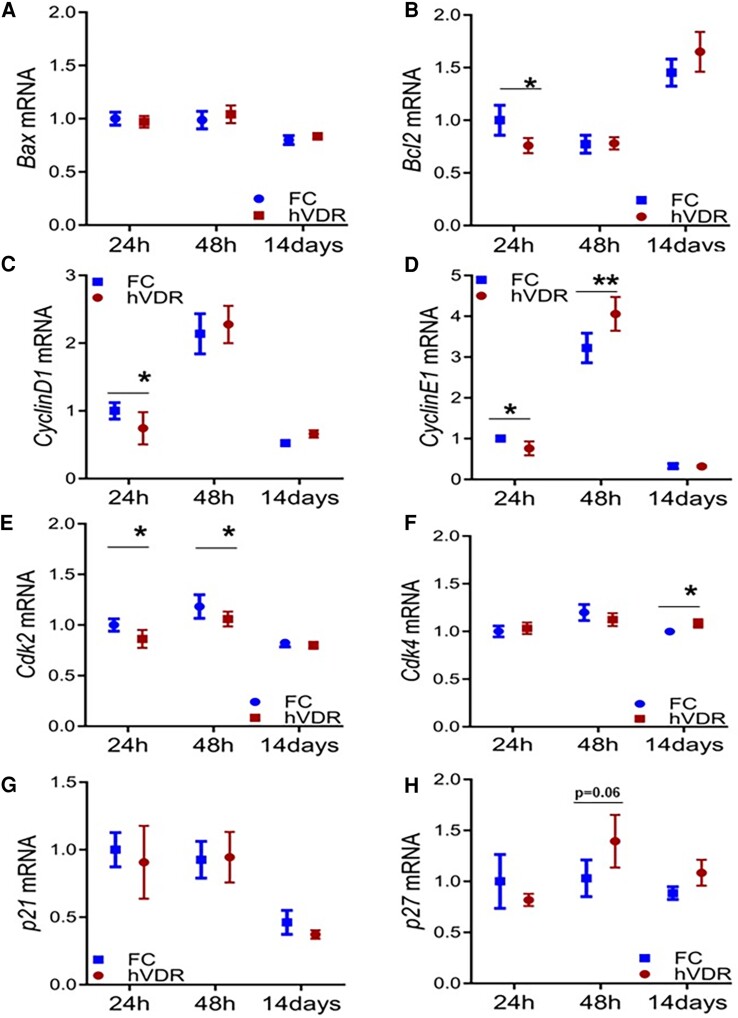
Gene expression in floxed control (FC) and hVDR mice, assessed by real-time PCR. Data shown as fold change vs 24-hour FC mice. All values corrected for expression of the housekeeping gene *Cyclophilin*, which did not differ between groups. (A) *Bax* mRNA. (B) *Bcl2* mRNA. (C) *Cylcin D1* mRNA. (D) *Cyclin E1* mRNA. (E) *Cdk2* mRNA. (F) *Cdk4* mRNA. (G) *p21* mRNA. (H) *p27* mRNA *P < .05 for indicated comparison (corrected for multiple comparisons).

VDR is a transcription factor, so next we assessed gene expression at 48 hours after partial hepatectomy by real-time PCR. We examined expression of pro-apoptotic *Bax* (Fig. 4A), which showed no significant differences between genotypes. *Bax* expression was lower at 14 days in both groups than at earlier times (*P* < .01 vs day 1 and vs day 2). Expression of antiapoptotic *Bcl2* was significantly lower in hVDR mice 1 day after surgery and did not differ significantly between genotypes at later times (Fig. 4B). *Bcl2* expression was significantly increased at 14 days compared to the earlier times but without difference between the genotypes.

Compared to FC, hVDR mice have significantly altered expression of genes important for cell cycle progression. There is decreased expression of *cyclin D1* at 1 day in hVDR vs FC mice (*P* < .05, Fig. 4C). At day 2, consistent with the greater Ki67 staining, there is increased *cyclin D1* mRNA in both genotypes, which returns to baseline by 14 days.

At 2 days, *cyclin E1* increases in both groups, and was greater in hVDR mice. hVDR mice had lower *cyclin E1* 24 hours after PHx (Fig. 4D) and higher levels at 2 days after PHx. By 14 days postsurgery, the *cyclin E* levels have reduced to below those seen at day 1 in both genotypes. Cyclin-dependent kinase 2 (*Cdk2*) was significantly altered by both time and genotype (Fig. 4E) with decreased expression in hVDR mice at days 1 and 2. Expression of *Cdk4* was significantly higher in hVDR mice at 14 days without differences between genotypes at other time points.

No significant changes in expression of the cell-cycle inhibitor *p21* were seen (Fig. 4G). The expression of the cell-cycle inhibitor *p27* showed a trend toward higher expression at 48 hours in hVDR mice when Ki67 staining was lower but did not achieve statistical significance (*P* = .06) (Fig. 4H).

**Figure 5.**
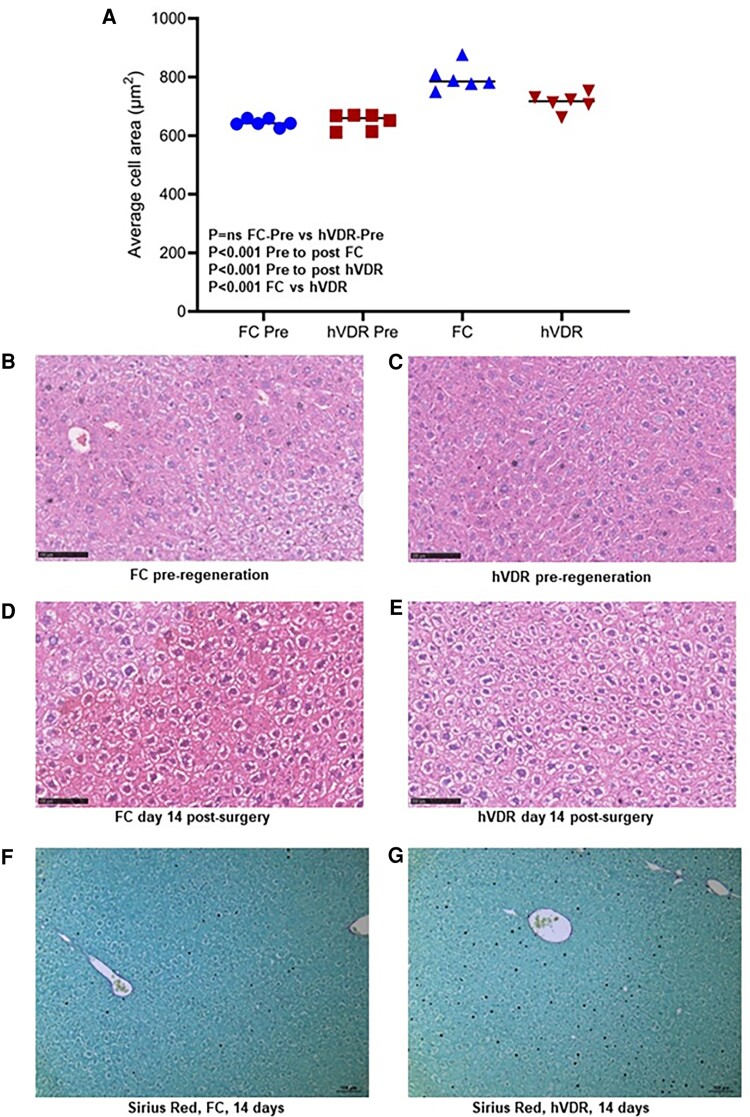
Histological changes in floxed control (FC) and hVDR mice. (A) Quantification of cell size in presurgery and day 14 partial hepatectomy mice, in 10 separated slides from each of 6 mice per group, analyzed by ANOVArm, *P* values corrected for multiple comparisons. Data show average per mouse (B) and (C) hematoxylin and eosin (H&E) in FC and hVDR mice (D) and (E) H&E in FC and hVDR mice at day 14. (F and G) Sirius Red in day 14 FC and hVDR mice.

We have previously reported alterations in individual cell size in muscle with VDR deletion (5, 17). We examined cell size in livers of FC and hVDR mice. There were no differences in cell size in the baseline liver resected on the day of partial hepatectomy, quantified in Fig. 5A, representative images Fig. 5B and 5C. However, after 2/3 hepatectomy, control mice had a substantial increase in average cell cross-sectional area at 14 days (23%, *P* < .0001, Fig. 5D). The hVDR mice also had a significant increase (*P* < .001, Fig. 5E) but the increase was less than 50% of the increase seen in FC mice at 11%. There was no difference in liver fibrosis as assessed by Sirius Red staining at 14 days (Fig. 5F and 5G).

**Figure 6.**
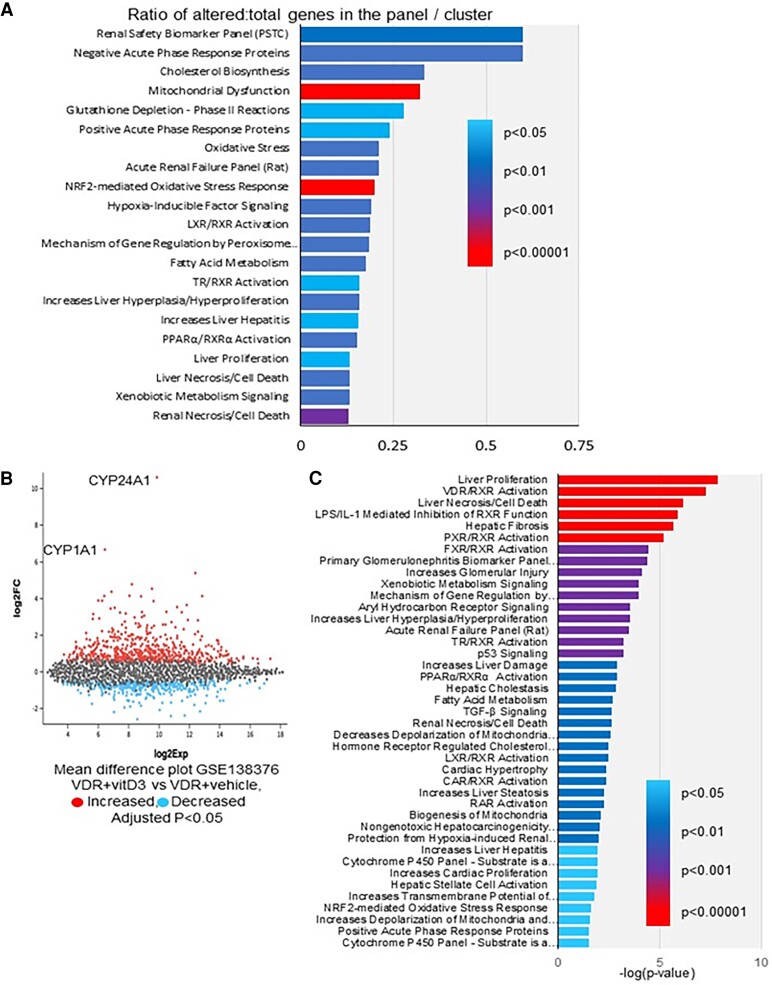
Gene expression profiling. (A) Patterns of gene expression changes in vitamin D-treated vs control-treated primary rat hepatocytes. (B) Analysis of publicly available dataset from Bozic et al (15). Mean difference plot for expression profiles of vitamin D vs control-treated hepatocytes. Blue symbols indicate genes decreased by vitamin D and red symbols indicate genes with increased expression after vitamin D. (C) Analysis of the same dataset showing overrepresented pathways regulated by vitamin D.

With significant changes in hepatocyte proliferation, bile acids, cell size, and final liver weight, we directly assessed the effects of treatment with vitamin D on cultured primary rat hepatocytes by profiling gene expression with and without vitamin D treatment (Fig. 6A). Vitamin D signaling is known to stimulate expression of the gene encoding 24-hydroxylase, which inactivates vitamin D. In primary hepatocytes, *Cyp24A1* mRNA was significantly increased by vitamin D treatment (2.48-fold, *P* < 5E^−35^), demonstrating capacity for vitamin D signaling.

Consistent with the effects in vivo with hepatocyte VDR deletion, genes involved in liver proliferation, hepatitis, liver hyperplasia/hyperproliferation, and liver necrosis/cell death pathways were overrepresented in genes that were significantly altered by vitamin D treatment (Fig. 6A).

We then conducted a manual analysis of the set called liver cell proliferation, cross-referencing that with the VDR chromatin immunoprecipitation sequencing data set GSE69179. Of the 24 genes in the liver cell proliferation set, 20 have VDR binding sites, mostly in the promoter region, as described in Table 2.

**Table 2.** Chromatin immunoprecipitation-sequencing VDR binding sites in the liver proliferation gene set

Gene	VDR binding	Location
AHR	Yes	Promoter and intron 1
C3	Yes	5′ flanking region
CEBPB	Yes	5′ flanking region and promoter
CTNNB1	Yes	Promoter and intron 1
CTSB	Yes	Promoter and intron 1
DICER1	Yes	Promoter and intron 1
EDNRB	No	
FADD	Yes	Promoter, exon 1, and intron 1
FBXL5	Yes	5′ flanking region
IL1RN	No	
ITGB1	Yes	Promoter
JUND	Yes	Promoter
LIMS1	Yes	Promoter
NCF1	Yes	5′ flanking region
ODC1	Yes	Promoter
OSMR	No	
PIM3	Yes	Promoter
PTPN6	Yes	Promoter
SLC20A1	Yes	Promoter
SPP1	No	
STAT1	Yes	5′ flanking region and promoter
TGFA	Yes	Promoter and intron 2
TNFRSF12A	Yes	Promoter and exon 3
TNFRSF1A	Yes	5′ flanking region, promoter and intron 1

We searched public databases for experiments examining effects of vitamin D in hepatocytes. Bozic et al tested cultured hepatoma cells, transfected with VDR (15). Analysis of their data shows a similar pattern of gene expression changes with a significant upregulation of Cyp24A1 (Fig. 6B) and liver proliferation and hyperplasia clusters in pathway analysis (Fig. 6C).

## Discussion

Liver regeneration occurs predominantly via proliferation of hepatocytes and it is reviewed in (18, 19). The latter paper also specifically reviews the 2/3 partial hepatectomy model (19).

In these studies we interrogated the role of vitamin D signaling in hepatocytes on regeneration of the liver. There was impaired cell proliferation in hVDR mice, accompanied by reduced expression of the antiapoptotic gene *Bcl2*, and reduced expression of cell-cycle genes *cyclin D1, cyclin E1,* and *cyclin-dependent kinase 2 (Cdk2).* There was also a tendency to increased expression of the cell-cycle inhibitor *p27.* This demonstrates that hepatocyte VDR is important for normal cell-cycle progression during liver regeneration. As well as reduced weight of the regenerated livers, liver function was abnormal in hVDR mice. This was assessed by profiling bile acids in floxed control and hVDR animals. Serum bile acids reflect enterohepatically recirculated bile acids that failed to be cleared by the liver. Notably, mice lacking hepatocyte VDR had significant increases in circulating conjugated bile acids after partial hepatectomy, consistent with failure to normalize hepatic function by the 14-day time point tested.

An important limitation of these studies is that the earlier time points (1 and 2 days) were carried out in younger mice. It is possible that this may have masked some early changes because young mice are known to have a greater regenerative capacity.

In rats, treatment with active vitamin D (1,25-dihydroxy-vitamin D) improves liver regeneration compared to regrowth observed in matching D-deficient animal (20). In that paper, Ethier et al showed that 50% liver regeneration was achieved by 50.6 hours in 1,25-dihydroxy-vitamin D treated rats compared to 70.8 hours in D-deficient animals. There was little effect of vitamin D treatment in animals without underlying vitamin D deficiency. Unavoidably, in a vitamin D deficiency/D treatment model, all the cells in the animal are affected by vitamin D, preventing the delineation of the role of VDR in hepatocytes on liver regeneration vs other cell types.

We did not identify any previous reports examining liver regeneration in whole-body VDR null mice. However, we have previously reported that by 6 months of age, some whole-body VDR null mice develop spontaneous liver cirrhosis (3). Again, this model does not distinguish the effects of VDR in hepatocytes specifically. The role of hepatocyte VDR in nonalcoholic steatohepatitis was examined by Zhang et al (21), who identified that hepatocyte VDR deletion potentiated diet-induced hepatosteatosis. After a high-fat diet, hVDR mice had poorer glucose tolerance and insulin sensitivity (21). These effects were not ameliorated by vitamin D treatment.

Partial hepatectomy increases bile acid flux in the remaining liver, which generates either a relative or absolute overload of bile acids. VDR in the cell membrane of hepatocytes can be activated by bile acids, triggering a signaling cascade that inhibits bile acid synthesis (22). This includes inhibition of *Cyp7a1* mRNA expression. Cyp7a1 is the rate-limiting enzyme in overall synthesis of bile acids (23).

Some bile acids are able to stimulate hepatocyte proliferation (16, 24). Huang et al reported that liver regeneration could be significantly enhanced by a relatively low concentration of cholic acid (CA), 0.2% in the diet (25). A similar effect was observed by Barone et al, in which a diet with 5% cholic acid increased liver size by almost 30% (24). Consistent with the ability of selected bile acids to stimulate hepatocyte proliferation, decreasing bile acids using cholestyramine significantly inhibited liver regeneration.

As discussed previously, the higher levels of some bile acids seen in hVDR mice after partial hepatectomy (Fig. 3G and 3H) suggest ongoing liver dysfunction. FXR signaling is important for hepatocyte proliferation (26). Chenodeoxycholate is a potent ligand of FXR (27) and increases liver regeneration. It is thus noteworthy that it was increased after partial hepatectomy in the control mice, but not in hVDR animals. Failure to increase chenodeoxycholate may have contributed to the reduced liver weight in hVDR mice.

We have previously reported that vitamin D and VDR are important for myocyte size and muscle function (5, 17, 28). Whole-body VDR null mice have smaller muscles and smaller myocytes (17). Surprisingly, mice with myocyte vitamin D receptor deletion also had smaller muscles but with larger myocytes (5). We assessed hepatocyte size and found that, at baseline, there was no difference in average cell cross-sectional area. There was a substantial increase in average cell area after PHx in control animals, but the increase was blunted by more than 50% in the hVDRs. This decrease in cell size may explain the difference in final liver weight at study completion.

Consistent with the role of VDR as a transcription factor, profiling of vitamin D-induced gene expression pattern changes in hepatocytes demonstrated that vitamin D regulates many components of hepatocyte proliferation, cell survival, and RXR pathways. VDR transcriptional activities are mediated by the heterodimeric complex with RXR (29). Genes involved in acute phase response and oxidative stress were also enriched among vitamin D regulated genes. It is interesting to note that there were only minimal changes in hVDR mice before PHx. This is consistent with the low expression of VDR in unstressed hepatocytes and suggests that the major role of VDR in hepatocytes may be in aiding recovery after injury or insult.

In these studies, no increase in liver fibrosis was observed. This is consistent with what we know about hepatic stellate cells being the primary drivers of this process (3, 30, 31). Stellate cells would only be indirectly affected by hepatocyte VDR deletion. However, in the clinical setting, both hepatocytes and stellate cells would be exposed to the prevailing vitamin D levels. Therefore, deficiency of vitamin D would promote hepatic fibrogenesis and inhibit hepatocyte regeneration.

Taken together, these results show that hepatocyte VDR signaling promotes liver regeneration and that deficiency of VDR in hepatocytes impairs this process. The mechanisms appear to be VDR-mediated regulation of many components of the cell cycle, and potentially a failure to upregulate chenodeoxycholate which also stimulates hepatocyte proliferation.

These findings are consistent with prevailing knowledge that the outcomes for people with vitamin D deficiency across a range of human liver diseases is poorer (1). Patients with chronic liver disease should avoid vitamin D deficiency to improve long-term hepatic outcomes especially where there is ongoing infectious, drug or alcohol-related insult to the liver.

## Data Availability

Data including gene expression profiling will be made publicly available after acceptance at: Partial hepatectomy files (figshare.com), DOI:10.6084/m9.figshare.25203098.

## Abbreviations

ALT: alanine aminotransferase
AST: aspartate aminotransferase
CDCA: chenodeoxycholate
FC: floxed control
FXR: farnesoid X receptor
hVDR: hepatocyte VDR null
PHx: partial hepatectomy
VDR: vitamin D receptor
